# Identifying the Critical Domain of LL-37 Involved in Mediating Neutrophil Activation in the Presence of Influenza Virus: Functional and Structural Analysis

**DOI:** 10.1371/journal.pone.0133454

**Published:** 2015-08-26

**Authors:** Shweta Tripathi, Guangshun Wang, Mitchell White, Michael Rynkiewicz, Barbara Seaton, Kevan Hartshorn

**Affiliations:** 1 Boston University School of Medicine, Department of Medicine, Boston, MA, United States of America; 2 Department of Pathology and Microbiology, University of Nebraska Medical Center, Omaha, NE, United States of America; 3 Boston University School of Medicine, Department of Biophysics, Boston, MA, United States of America; Linneaus University, SWEDEN

## Abstract

The human cathelicidin LL-37 has been shown to play a role in host defense against influenza A viruses (IAV) through direct antiviral effects and through modulating inflammatory responses to infection. We recently showed that LL-37 increases neutrophil respiratory burst and neutrophil extracellular trap (NET) responses to IAV through engaging formyl peptide receptor 2 (FPR-2). In this paper we show that a fragment of LL-37, GI-20, which is composed of the central helical segment of the peptide, has similar effects as LL-37 on neutrophil activation. In addition to increasing respiratory burst and NET responses of the cells to IAV through an FPR-2 dependent mechanism, it reduces neutrophil IL-8 production to IAV (also like LL-37). The N-terminal fragment, LL-23, did not have similar effects. Both GI-20 and LL-37 increase neutrophil intracellular calcium levels and their ability to increase neutrophil activation responses was calcium dependent and partially inhibited by pertussis toxin. These studies show that the central helix of LL-37 retains the ability of LL-37 to modulate neutrophil responses through FPR-2. Based on our findings we developed a homology model of FPR-2 and performed docking experiments of LL-37 and GI-20 with the receptor.

## Introduction

Innate immunity plays a key role in the initial phase of the response to influenza A virus (IAV) infection [1]. Overall the innate response to IAV appears to be protective; however, some aspects of the response may contribute to inflammatory injury in the lung as well. There is strong evidence that antimicrobial peptides, including defensins and the cathelicidin, LL-37, contribute to the innate response to IAV. Antimicrobial peptides, including LL-37, have two modes of action in host defense: direct inhibition of infectivity of various pathogens and modulation of responses of various immune cells [2]. Defensins and LL-3 inhibit IAV in vitro [3–10]. Neutrophils are a major component of the early response to IAV in the respiratory tract and they likely serve as a source of defensins and LL-37 [11]. Respiratory epithelial cells also produce LL-37 and β-defensins in response to infection. Hence it is likely that these peptides are present in significant quantities during the early phase of IAV infection.

LL-37 has been shown to improve outcome of IAV infection in mice through inhibition of viral replication and reduction of virus-induced pro-inflammatory cytokine generation [12]. Upregulation of LL-37 expression by stimulation with LTB4 correlated with improved outcome of IAV infection in mice[13]. Defensins, retrocyclins and LL-37 all have been shown to modulate phagocyte responses to IAV and other pathogens [14]. LL-37 also causes secondary necrosis of apoptotic neutrophils [15]. This effect was found to be retained in the murine homologue of LL-37 (called CRAMP) and C-terminal but not N-terminal fragments of LL-37. Defensins have distinct mechanisms through which they modulate neutrophil responses compared to LL-37. Defensins aggregate IAV and increase IAV uptake by neutrophils while reducing neutrophil respiratory burst responses to the virus [16]. In contrast, LL-37 does not aggregate IAV or increase neutrophil uptake of the virus, but it does increase the virus induced neutrophil respiratory burst response [17]. LL-37 also increases neutrophil extracellular trap (NET) formation in response to IAV [17]. LL-37 inhibits pro-inflammatory phagocyte cytokine responses to various stimuli including LPS and IAV [17, 18]. We found specifically that LL-37 reduced neutrophil IL-8 production in response to IAV. There is evidence for several specific receptors for LL-37 on immune cells, including formyl peptide receptor 2 (FPR2; formerly referred to as formyl peptide receptor like 1 or FPRL1) on neutrophils, monocytes and lymphocytes [19], GAPDH in the cytoplasm of monocytes [20], CXCR2 on neutrophils [21], epidermal growth factor receptor on epithelial cells [22], and P2X7 in neutrophils and other cells [23]. We found that the ability of LL-37 to increase neutrophil respiratory burst responses and NET formation in response to IAV is counteracted by blockade of FPR2 using the specific inhibitor WRW4 [17].

There are naturally occurring fragments of LL-37 produced in tissue sites [24] and there have been efforts to determine which fragments of the protein retain anti-microbial and/or immune modulatory activities [25–28]. We have studied various synthesized fragments of LL-37 that have been reported to have increased anti-bacterial or antiviral activity [29, 30]. In the current study we compare the ability of biologically active fragments of LL-37 to modulate neutrophil responses to IAV with that of full length LL-37. Of the fragments tested in the current study, LL-23 is known to be a naturally occurring one, whereas the others are not known to occur naturally. One of the fragments, GI-20, which is composed of the central helix of LL-37 was of particular interest since it has been shown to be entirely helical in structure and to have the highest activity against HIV [31]. In addition, we recently showed that GI-20 retains the antiviral activity of LL-37 for IAV and also can inhibit pandemic IAV of 2009, whereas full length LL-37 cannot [32]. In this paper we show that GI-20 has very similar effects on neutrophil responses to IAV and, like LL-37, appeared to engage FPR2 on neutrophils. We also analyzed further the mechanisms through which LL-37 and GI-20 increase neutrophil respiratory burst responses to IAV.

## Methods

### Ethics Statement

All human subjects research in this study was approved by the Institutional Review Board of Boston University School of Medicine. A written consent form was specifically approved by the Institutional Review Board and signed by all participants and copies were kept in a locked cabinet only accessible to approved personnel involved in the study.

### Virus Preparations

Philippines 82/H3N2 (Phil82) strain was kindly provided by Dr. E. Margot Anders (Univ. of Melbourne, Melbourne, Australia) and grown in the chorioallantoic fluid of ten day old chicken eggs and purified on a discontinuous sucrose gradient as previously described [33]. The virus was dialyzed against phosphate buffer saline (PBS) to remove sucrose, aliquoted and stored at -80°C until needed. Post thawing the viral stocks contained ~5x10^8^ infectious focus forming units/ml.

### LL-37 and other reagents

LL-37 and the FK-13 and KR-12 fragments were purchased from Phoenix Pharmaceuticals, Burlingame, CA, USA. The scrambled LL-37 was purchased from Abgent Inc. LL-23, LL-23V9 and the central fragment of LL-37 (also referred to as GI-20) were developed as described in Dr Wang’s laboratory. HNP1 and HNP-2 were purchased from Bachem (Torrance, CA). The formyl peptide receptor like (FPRL) agonist peptide WKYMV and WRW4 blocking peptide were purchased from Phoenix Pharmaceuticals, Burlingame, CA, USA Phorbol myristate acetate (PMA), BAPTA and BAPTA-AM and Diphenyleneiodonium (DPI) was purchased from Sigma-Aldrich, St Louis, MO.

### Human Neutrophil Preparation

Neutrophils from healthy volunteers were isolated to > 95% purity by using dextran precipitation, followed by Ficoll-Paque gradient separation for the separation of mononuclear cells (layering above the Ficoll-Paque) and neutrophils (below the Ficoll-Paque). The neutrophils were purified further by hypotonic lysis to eliminate any contaminating erythrocytes, as previously described [33]. Cell viability was determined to be >98% by trypan blue staining. The isolated neutrophils were resuspended at the appropriate concentrations in PBS and used within 2 hours. Neutrophil collection was done with written informed consent as approved by the Institutional Review Board of Boston University School of Medicine. The written consent form was specifically approved by the Institutional Review Board and copies were kept in a locked cabinet only accessible to approved personnel involved in the study. To remove extracellular and intracellular calcium for certain assays, the neutrophils were incubated with BAPTA or BAPTA-AM at final concentrations of 100 μM for 45 min at 37°C, followed by re-suspension of the cells in fresh buffer for assays of H_2_O_2_ production or NET formation.

### Measurement of IAV uptake by neutrophils

Fluorescein isothiocyanate (FITC)-labeled IAV (Phil82 strain) was prepared and uptake of virus by neutrophils was measured by flow cytometry as described [34]. In brief, IAV was treated with various doses of LL-37 for 30 min at 37°C. Then it was incubated with cells for 45 minutes at 37°C in presence of control buffer. Trypan blue (0.2 mg/ml) was added to these samples to quench extracellular fluorescence. Following washing, the neutrophils were fixed with 1% paraformaldehyde and neutrophil associated fluorescence was measured using flow cytometry. The mean cell fluorescence (>1000 cells counted per sample) was measured.

### Measurement of neutrophil H_2_O_2_ production

H_2_O_2_ production was measured by assessing reduction in scopoletin fluorescence as previously described [35]. Measurements were made using a POLARstar OPTIMA fluorescent plate reader (BMG Labtech, Durham NC). WKYMV peptide was used at a concentarion of 10 nM. For WRW4 treatment, neutrophils were incubated with 20 μM WRW4 for 5 mins at room temperature and then used for experiment.

### Assessment of NET formation

For NET experiments, neutrophils were resuspended in PBS supplemented with Ca^2+^ and Mg^2+^ and allowed to adhere on Poly-L-Lysine coated 96 well plates or on glass bottom culture dishes (MatTek corporation, Ashland, MA) for 1 hr in a CO_2_ incubator. After 1 hr, unadhered cells were removed and adhered cells were incubated for 3 hrs in a CO_2_ incubator with Phil82 virus with or without pre-incubation (30 min, 37°C) with LL-37. For quantitative assessment of NETs, 5 μM Sytox green (Invitrogen, NY, USA) was added to samples on 96 well plate after the 3 hr incubation period and the plate was read on POLARstar OPTIMA fluorescent plate reader (BMG Labtech, Durham NC). Immediately after reading the plate, the plate was also photographed on a fluorescent microscope. Neutrophil NET formation was also assessed using confocal microscopy. For confocal experiments, PMNs were adhered on poly-L-lysine coated glass bottom culture dishes and incubated for 3 hrs with Phil82 virus and LL-37 followed by fixation with 1% paraformaldehyde. Wheat germ agglutinin (WGA)-Oregon Green 488 (4 μg/ml) and DAPI 350 (Invitrogen, USA) were used to stain the cell membrane and nucleus respectively. The virus was Alexa Flour 594 labeled using a kit (Invitrogen, USA) as per manufactures instructions. Confocal pictures were taken at Zeiss LSM510 (LSEB) on 100x resolution.

### Measurement of IL-8 production by neutrophils

For testing the effect of peptides on IL-8 production, neutrophils were re-suspended in PBS and infected with Phil82 virus. The virus was pre-incubated peptides for 30 min at 37°C prior to addition to neutrophils. The neutrophils were then treated with these virus (+/- peptide) preparations for 45 min in a CO_2_ incubator. After this the cells were pelleted and then re-suspended and cultured in RPMI with 10% heat inactivated autologous serum for 20 hrs. After 20 hrs, the supernatant was collected and assayed for IL-8 using a commercially available ELISA kit (BD Biosciences, San Diego, CA) according to the manufacturer’s instructions. Cells without any stimulus or with LPS as stimulus (100 ng per 5x10^5^ cells, Sigma-Aldrich, St Louis, MO) were used as negative and positive controls in the experiment. As additional controls other cultures of the cells were treated with heat-inactivated IAV or UV-irradiated IAV [36].

### Measurement of intracellular calcium responses of neutrophils

Intracellular calcium responses of neutrophils was measured using the Fluo-4 NW calcium assay kit (Molecular Probes, Invitrogen). This assay has the advantage that it is not necessary to wash cells after addition of the Fluo-4 dye. Briefly cells are incubated with assay buffer (Hank’s balanced salt solution with 20 mM HEPES) for sixty minutes at 37°C at a concentration of 2x10^6^ cells per ml in 96 well black plates (50 μl/well). The dye solution is prepared separately and includes also probenicid (final concentration 5 mM) to reduce extrusion of the dye outside of the cells. Fifty μl of the dye solution is added to all wells and there is a further 30 min incubation at 37°C. After this stimuli (fMLP, peptides alone, or IAV with or without peptides) are added and fluorescence assessed at excitation of 494 nm and emission of 516 nm using a POLARstar OPTIMA fluorescent plate reader (BMG Labtech, Durham NC).

### Homology model of formyl peptide receptor 2

The homology model of the formyl peptide receptor 2 (FPR2) was built using the I-TASSER server [37]. The templates used for the building included the CCR5 chemokine receptor [38], the human delta opioid receptor [39], and the human β2-adrenergic G protein-coupled receptor [40]. The 4 models of the NMR structure of LL-37 [28] were then docked to the FPR2 model using the ClusPro 2.0 server [41].

## Results

### The central fragment of LL-37, GI-20, potentiates neutrophil respiratory burst response to IAV but N-terminal fragments and CRAMP do not

**Fig 1:**
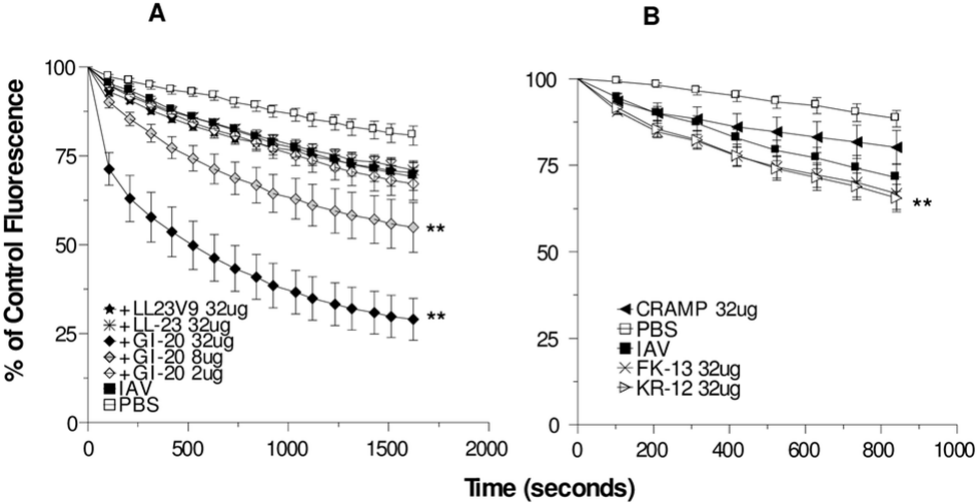
Neutrophil H2O2 production in response to IAV or IAV + LL-37derived peptides–Neutrophils were treated with control buffer, IAV, or combinations of IAV and GI-20, LL23, or LL23V9 (panel A) or CRAMP, KR-12 or LK-13 (panel B) and H2O2 responses were measured by the fluorescent scopoletin assay. Decrease in scopoletin fluorescence corresponds to H_2_O_2_ production. A dose response using GI-20 was performed (doses as indicated). For the other peptides 32 μg/ml was used. IAV alone elicited increased H_2_O_2_ generation as compared to control buffer. Pre-incubation of IAV with GI-20 caused significant further increases in H_2_O_2_ generation in a dose related manner compared to IAV alone (** indicates p<0.05 compared with IAV alone). KR-12 caused slight but statistically significant increase in H_2_O_2_ generation compared with IAV alone. Results are mean ± SEM of 3–5 experiments using separate neutrophil donors.

As shown in Fig 1A, IAV alone causes a modest, but significant increase in neutrophil H_2_O_2_ production as previously reported [42]. Pre-incubation of IAV with GI-20 caused a marked, dose-dependent increase in neutrophil H_2_O_2_ response to IAV. These results were very similar to those we have reported with full length LL-37 [43]. We have previously reported that LL-37 on it’s own does not trigger any H_2_O_2_ production from neutrophils [43]. For this study we found that GI-20, LL-23 or LL23V9, did not trigger any greater H_2_O_2_ production than PBS buffer alone (data not shown; n = 3). We also tested the activity of a several other fragments of LL-37, including the N-terminal fragments, LL-23 and LL-23V9, and the shorter central fragments, FK-13 and KR-12. LL23V9 was synthesized by Dr. Wang in order to form a continuous hydrophobic surface on the peptide. It has been shown to have increased anti-bacterial activity compared to the native fragment, LL-23 [27]. The LL-23 and LL-23V9 fragments did not increase neutrophil H_2_O_2_ production in response to IAV. The combination of KR-12 and IAV caused a slight increase in H_2_O_2_ production compared to IAV alone (Fig 1B). CRAMP at a concentration of 32 μg/ml did not significantly increase H_2_O_2_ production.

Neither GI-20, nor the other peptides, caused an increase in viral uptake by neutrophils (data not shown). This was tested using fluorescent labeled IAV and flow cytometry. LL-37 also did not have this effect in prior studies [43].

**Fig 2:**
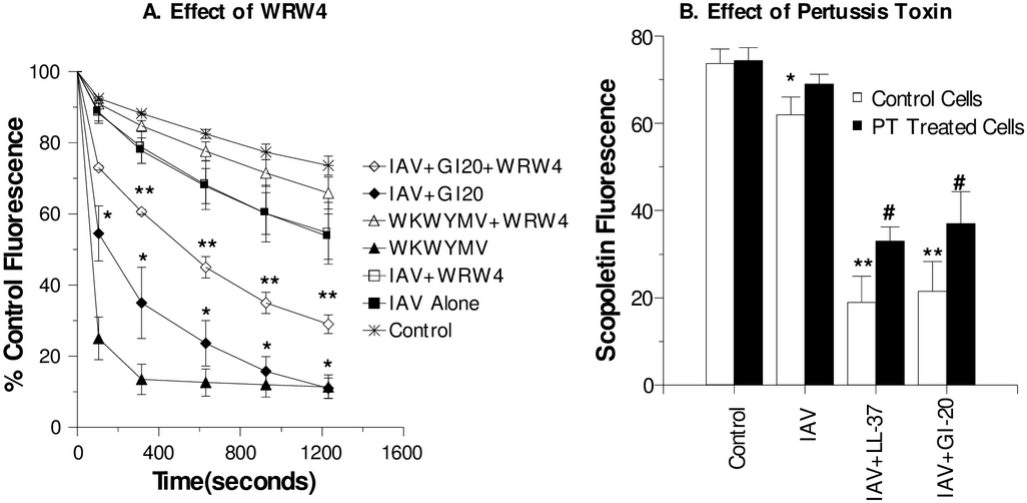
Effect of FPRL blockade on H_2_O_2_ generation in response to IAV or IAV + GI-20 –Experiments were performed as in Fig 1. In panel A, neutrophils were stimulated with IAV, IAV+GI-20 (32μg/ml), or the WKYMV peptide alone or combined with the WRW4 blocking peptide. Neutrophils treated with PBS buffer alone (control) are shown for comparison). WRW4 markedly reduced the response to WKYMV (p<0.001) but not to IAV. When IAV was pre-treated with GI-20, a significantly increased response was observed compared to IAV alone (p<0.001; indicated by *). This response was significantly reduced by WRW4 (p<0.01; indicated by **). Results are mean ± SEM of 3–5 experiments using separate neutrophil donors. In panel B, neutrophils were pre-incubated for 90 min with pertussis toxin or control buffer and then stimulated with either control buffer alone, IAV alone, or IAV that had been pre-treated with LL-37 or GI-20 (32μg/ml). Decrease in scopoletin fluorescence indicates H_2_O_2_ generation. * indicates significant reduction of scopoletin fluorescence for IAV treated cells as compared to control buffer alone (p<0.05). ** indicates significantly greater decrease when comparing IAV+LL-37 or IAV+GI-20 when compared to IAV alone (p<0.05 by ANOVA). # indicates where pertussis toxin significantly inhibited the scopoletin response (p<0.05 by ANOVA).

Pre-incubation of neutrophils with WRW4 (and FPR-2 blocker) inhibited H_2_O_2_ production in response to the WKYMV peptide but did not reduce the response to IAV alone (Fig 2A). H_2_O_2_ production triggered by the combination of GI-20 and IAV was partially inhibited by WRW4 (Fig 2A). Pre-incubation of the neutrophils with pertussis toxin (PT) also partially reduced the response to IAV which had been pre-incubated with either LL-37 or GI-20 (Fig 2B). In Fig 2B the maximal reduction in scopoletin fluorescence (the indicator of H_2_O_2_ production) after 15 min of incubation with IAV are shown.

**Fig 3:**
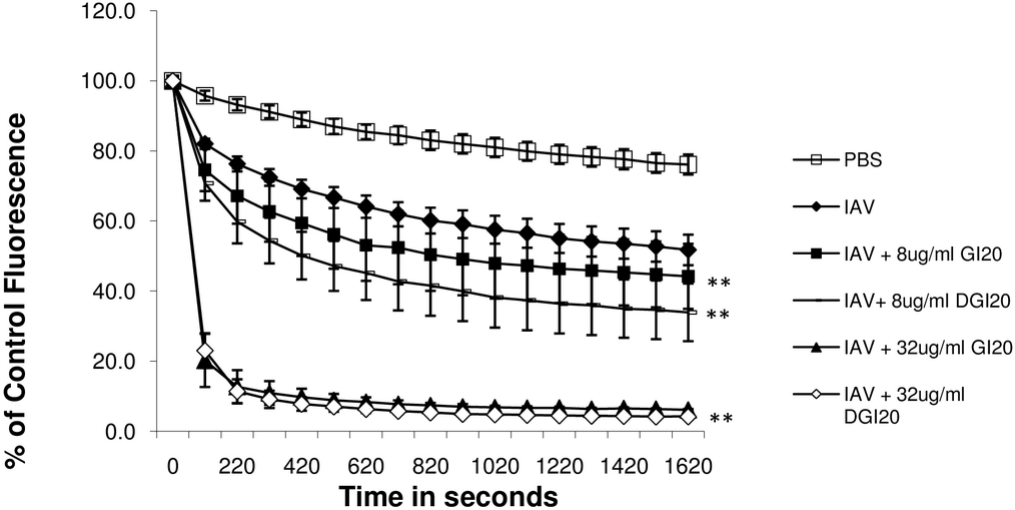
Comparison of effects of L- and D-isomers of GI-20 on neutrophil H_2_O_2_ generation in response to IAV—Experiments were performed as in Fig 1. Neutrophils were treated with control buffer, IAV, or combinations of IAV and the L- or D-isomers of GI-20. Pre-incubation of IAV with either form of GI-20 caused significant increases in H_2_O_2_ generation in a dose related manner compared to IAV alone (** indicates p<0.05 compared with IAV alone). There was no significant difference between the effects of L- or D-isomers of GI-20 in these assays. Results are mean ± SEM of 4 experiments using separate neutrophil donors.

In order to further probe the structural requirements of receptor activity we compared the ability of the D- and L-isomers of GI-20 to potentiate respiratory burst responses. As shown in Fig 3, the D-isomer forms of GI-20 had nearly identical ability to increase responses as the L-isomer. Neither isomer of GI-20 caused neutrophil production of H_2_O_2_ on its own in absence of virus (data not shown).

### IAV induced neutrophil extracellular trap (NET) formation is increased by pre-incubation of the virus with GI-20

**Fig 4:**
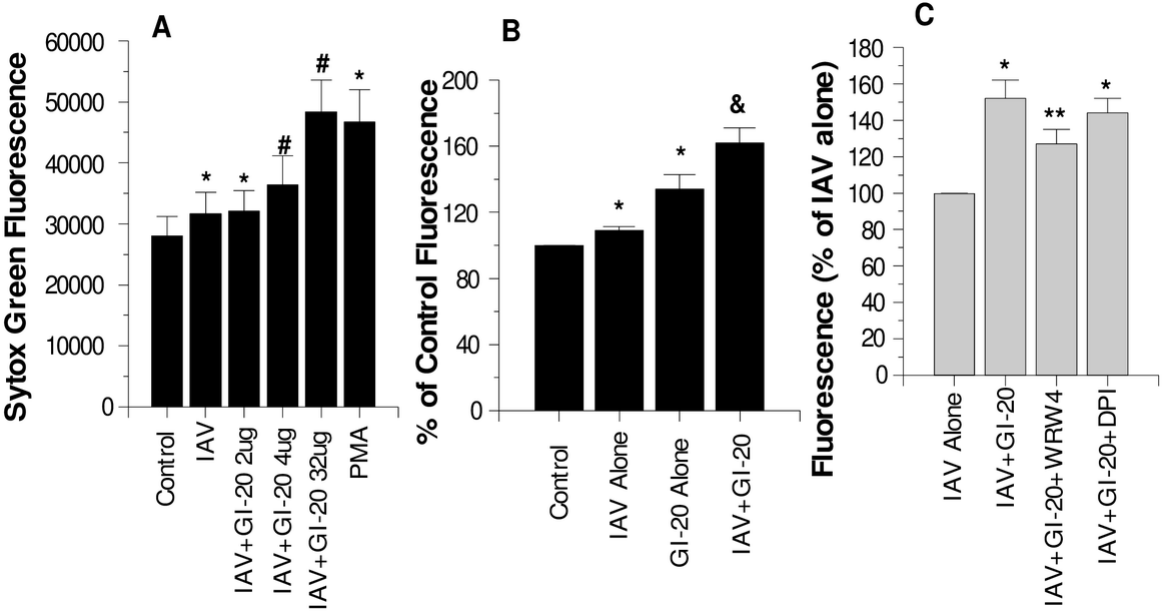
Effects of GI-20 on neutrophil NET formation–Panel A shows Sytox green fluorescence measurements of neutrophils treated with control buffer, PMA, IAV, or IAV pre-treated with the indicated concentrations/ml of GI-20. IAV alone or in combination with GI-20 significantly increased NET formation as compared to PBS treated cells (* indicates p<0.05 compared with control). GI-20 caused significant further increases in NET formation compared to IAV alone where indicated by #). Panel B compares the effects of IAV alone, GI-20 alone (at the highest concentration tested in panel A) and the combination of IAV and GI-20 on NET responses. GI-20 did cause increased NET formation compared to PBS alone. However, the combination of IAV and GI-20 caused significantly more NET formation than either stimulus alone (p<0.005 by ANOVA; n = 7). Panel C shows the effects of WRW4 (FPR2 blocker) or DPI (NADPH oxidase blocker) on NET responses to IAV or IAV+GI-20. WRW4 significantly decreased the response to the combination of GI-20 with IAV (** indicates p<0.01), but DPI did not. Results are representative of three experiments with different neutrophil donors. * indicates where fluorescence was significantly increased (p<0.01) compared to control cells alone. # indicates where addition of GI-20 caused significant increases in NET formation compared to IAV alone. & indicates where the combination of GI-20 and IAV caused significantly more NET formation than either GI-20 or IAV alone (p<0.005, assessed by ANOVA). ** indicates where WRW4 significantly reduced NET response to IAV+GI-20.

As previously reported, IAV alone induced NET formation by neutrophils [43]. As shown in Fig 4, GI-20 increased NET formation in response to IAV. GI-20 elicited NET formation on its own as well; however, significantly greater NET formation resulted from the combination of GI-20 and IAV than from either GI-20 or IAV alone. Fig 3B shows also that WDW4 reduced the NET response of neutrophils to the combination of IAV and GI-20. In contrast, DPI (inhibitor of the NADPH oxidase) did not reduce the response. These results are again similar to those obtained with LL-37 and IAV [43].

### Effects of GI-20 on neutrophil IL-8 production

**Fig 5:**
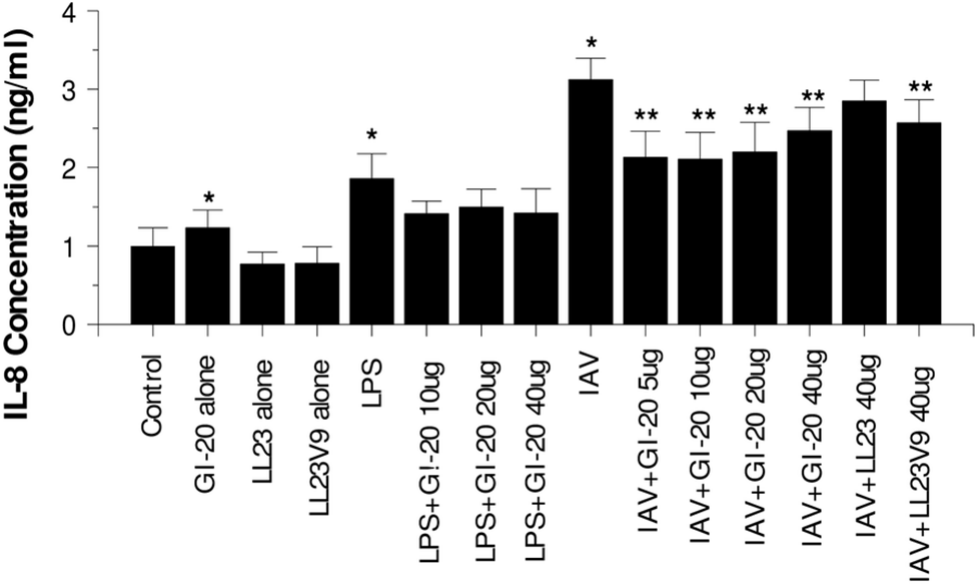
Effects of IAV or IAV plus GI-20, LL-23 or LL-23V9 on neutrophil IL-8 generation–Neutrophils were stimulated with either LPS, IAV (Phil82 strain), GI-20, LL-23 or LL-23V9 alone, or combinations of IAV or LPS with the peptides, and IL-8 production in the cell supernatants were measured 20 hrs later. LPS and IAV caused increased IL-8 production compared to control (untreated neutrophils) (indicated by *; p<0.01). GI-20 reduced IL-8 generation in response to IAV (indicated by ** for p<0.05). The peptides alone [except for the highest concentration of GI-20 (20 μg/ml)] did not increase IL-8 generation compared to control in the absence of LPS or IAV. GI-20 reduced responses to LPS but this was not statistically significant. Results are mean±SEM of 6 experiments with separate neutrophil donors. * indicates significant increase at p<0.05 compared to control ** indicates a significant decrease compared to IAV or LPS alone at p<0.05

We have reported that IAV strongly induces IL-8 production by neutrophils and that this response is reduced by pre-incubation of the virus with LL-37 [43]. Unlike the relatively immediate H_2_O_2_ response, IL-8 production occurs hours after incubation with IAV and peaks at 18 to 24 hours. As shown in Fig 5, pre-incubation of IAV with GI-20 inhibited IAV-induced IL-8 production after 18 hours of exposure to the virus. LL-23 did not have this effect. LL-23V9 did cause slight, but significant, reduction in the response. The effect of GI-20 was not dose related and in fact seemed to diminish at the highest concentration tested (40 μg/ml). This may in part reflect the fact that this concentration of GI-20 caused slight stimulation of IL-8 production in the absence of IAV. LPS triggered IL-8 production and pre-incubation of LPS with GI-20 showed a trend toward reducing this response but this was not statistically significant.

### Role of neutrophil intracellular calcium signaling in potentiation of neutrophil activation by LL-37 and GI-20

**Fig 6:**
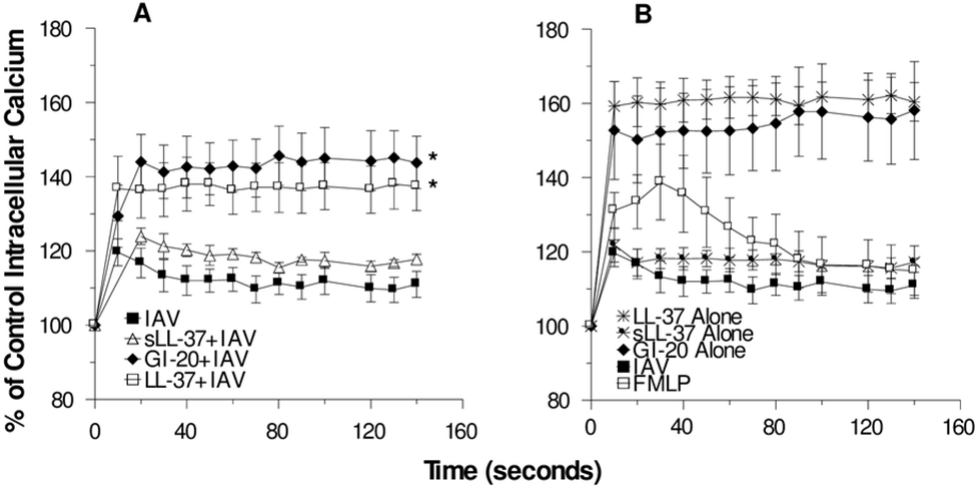
Calcium responses to IAV, fMLP, and LL-37 or GI-20 with or without IAV–Neutrophil intracellular calcium was assessed using…. In panel A the intracellular calcium fluxes resulting from incubation with IAV alone or IAV that was pre-incubated with either LL-37, sLL-37 or GI-20 (all at 32μg/ml) are shown. The rise in calcium of cells treated with IAV that was pre-incubated with LL-37 or GI-20 was significantly greater than that seen with IAV alone or IAV that was pre-incubated with sLL-37. In panel B the calcium responses to peptides alone are shown. The response to fMLP was included for comparative purposes. Results are mean±SEM of 4 to 5 experiments using different neutrophil donors.

LL-37 and GI-20 on their own caused marked and sustained elevation of neutrophil intracellular calcium levels as determined by Fluo-4 fluorescence (Fig 6A and 6B). Wan et al previously showed similar results for LL-37 [44]. As in our prior studies IAV or fMLP alone caused an initial rise in intracellular calcium which then returned at least partially to baseline (Fig 6A and 6B). When IAV was pre-treated with either LL-37 or GI-20 there was a greater rise in intracellular calcium as compared to IAV alone (Fig 6A).

**Fig 7:**
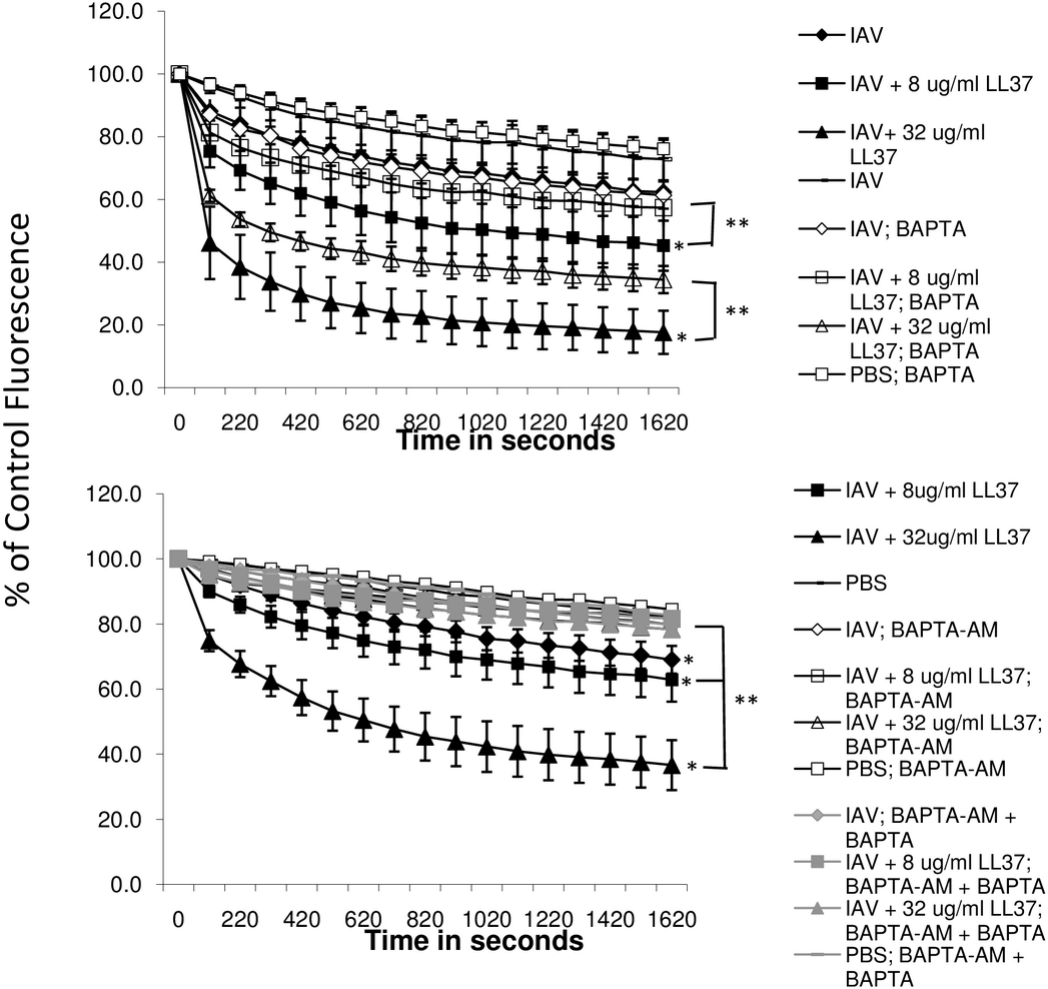
Effect of calcium chelation on H_2_O_2_ production in response to IAV or IAV+LL-37 –H_2_O_2_ production was measured as in Fig 1. Prior to the assay neutrophils were pre-incubated with BAPTA-AM or a combination of BAPTA-AM and BAPTA. IAV caused increased H_2_O_2_ production compared to PBS alone, and LL-37 caused dose related further increases of the response to IAV (* indicates p<0.01 for comparison of IAV vs. IAV+LL-37). Responses to IAV or to combinations of IAV with LL-37 were reduced to the level of PBS alone by BAPTA-AM or the combination of BAPTA-AM and BAPTA (p<0.05 for all comparisons of BAPTA treated cells and IAV or IAV+LL-37). Results represent mean±SEM of 5 experiments using separate neutrophil donors.

**Fig 8:**
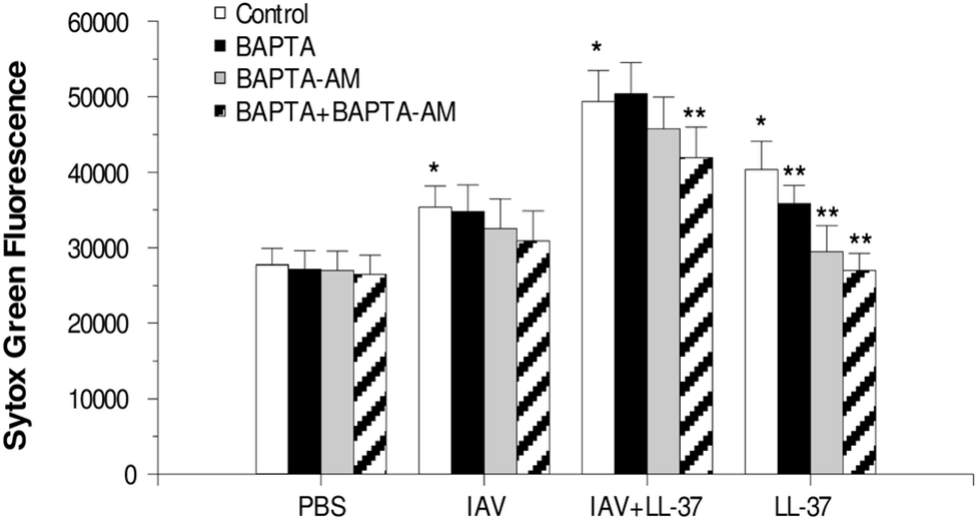
Effect of calcium chelation on NET response–Neutrophils were treated with either IAV alone (Phil82 strain) or IAV that had been pre-incubated with LL-37 (32 μg/ml). NET generation was measured as in Fig 3 using the Sytox green assay. Prior to treating the neutrophils with IAV, they were pre-incubated with either BAPTA, BAPTA-AM or both to chelate extra- and/or intra-cellular calcium. As in prior experiments, IAV increased NET response compared to PBS alone, and LL-37 significantly increased the NET response to IAV. LL-37 alone (at the highest concentration tested) did cause increased NET formation compared to PBS alone. However, the combination of IAV and GI-20 caused significantly more NET formation than either stimulus alone (p<0.05 by ANOVA). The combination of BAPTA and BAPTA-AM significantly decreased the NET response to LL-37 alone or the combination of IAV and LL-37 (** indicates p<0.05; n = 5 with separate neutrophil donors).

To determine the extent to which intracellular calcium contributed to respiratory burst and NET responses to IAV or IAV complexed with LL-37, we tested the effects of intra- and/or extra-cellular calcium chelation on the responses using BAPTA or BAPTA-AM. In these experiments IAV caused a slight elevation of H_2_O_2_ generation as compared to control buffer, and this was again increased when the virus was pre-incubated with LL-37. BAPTA chelates extracellular calcium while BAPTA-AM chelates intracellular calcium. BAPTA alone caused partial (but statistically significant) reduction in H_2_O_2_ responses to combinations of LL-37 with IAV (Fig 7A). This indicates a partial dependence of the enhanced response caused by LL-37 on extracellular calcium. The effects of intracellular calcium chelation were more pronounced. BAPTA-AM or the combination of BAPTA plus BAPTA-AM inhibited responses to IAV or IAV plus LL-37 to the level of control buffer (Fig 7B). The effects of BAPTA-AM on NET formation in response to IAV+LL-37 were less pronounced (Fig 8); however, the combination of BAPTA-AM and BAPTA together did significantly reduce NET formation in response to IAV+LL-37. LL-37 alone elicited NET formation to some extent in these experiments, although the degree of NET formation caused by the combination of IAV and LL-37 was significantly greater than for either LL-37 or IAV alone. Intracellular and extracellular calcium chelation with BAPTA-AM or BAPTA significantly reduced the NET response to LL-37 alone.

## Discussion

In addition to its action as an anti-microbial and anti-viral peptide, LL-37 has important immune-modulatory activities mediated through interaction with a variety of receptors on phagocytes and other cells. We have shown that the ability of LL-37 to markedly potentiate neutrophil respiratory burst and NET responses to IAV is mediated at least in part by FPR-2. In the current study we show that GI-20 has similar effects. These effects of GI-20 were largely mediated by FPR-2 since they were inhibited by the FPR-2 blocker WRW4 and partially pertussis toxin sensitive. We show as well that GI-20 reduces IL-8 production triggered by IAV in neutrophils (also like LL-37). In contrast, the N-terminal fragment LL-23 lacked these activities. GI-20 is composed of the extended central helix of LL-37. KR-12 and LK-13 have anti-bacterial activity and are also derived from the central helix of LL-37. They had only minimal ability to increase neutrophil respiratory burst response to IAV. Overall these findings indicate that GI-20 retains the ability to modulate neutrophil responses to IAV in a similar manner as LL-37 and is able, like LL-37, to engage FPR-2 on these cells, whereas N-terminal fragments of LL-37 do not.

Of interest, the murine cathelicidin, CRAMP, did not potentiate virus induced respiratory burst responses suggesting lack of interaction with human FPR-2. When complexed with LL-37, poly I:C is directed to a different endocytic pathway in human airway epithelial cells compared to poly I:C alone [45]. This re-direction is dependent on interaction with FPR-2 and CRAMP did not share this activity. Despite other similarities, therefore, CRAMP and LL-37 may have distinct interactions with FPR-2 or other receptors.

The ability of LL-37 and GI-20 to potentiate initial activation responses of neutrophils to IAV appears to be dependent their ability to elevate intracellular calcium levels. GI-20 caused a very similar strong and sustained rise in intracellular calcium as LL-37. Chelation of intracellular calcium fully abolished the respiratory burst response to IAV or IAV complexed with LL-37. Calcium chelation also reduced NET formation in response to IAV+LL-37, although the inhibition was not as complete as for respiratory burst responses. DPI, a strong inhibitor of respiratory burst responses of neutrophils mediated through the NADPH-oxidase (NOX), did not inhibit NET responses to IAV or IAV+LL-37 (as previously reported) or IAV+GI-20 (Fig 4B). These findings indicate that the ability of LL-37 to potentiate NET responses to IAV is not causally linked to respiratory burst activation. Further studies are underway to elucidate the mechanism of IAV or IAV+LL-37 NET formation. A recent study by Douda et al demonstrated a calcium activated, but NOX-independent, form of NETosis [46]. Such a pathway may be involved in IAV or IAV+LL-37 mediated NETosis.

**Fig 9:**
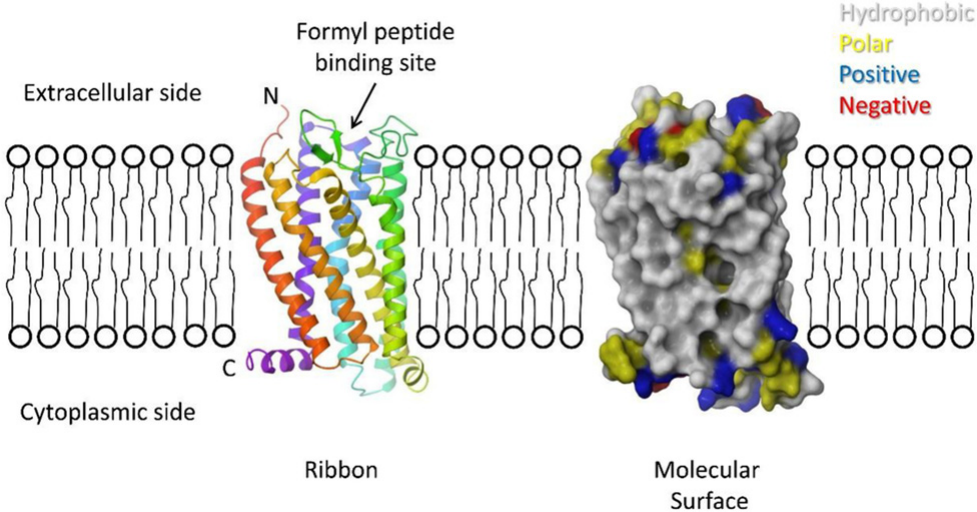
Homology model of structure of FPR-2 –The model was developed based on known structures of related seven transmembrane component, G-protein coupled receptors as described in Methods. On the left is a ribbon diagram of the model colored as a rainbow spectrum starting with red at the N-terminus and violet at the C-terminus, showing the seven transmembrane helices expected for this receptor. On the right is a molecular surface of the model colored by residue type–grey for hydrophobic residues, yellow for polar non-charged residues, blue for positive residues, and red for negative residues. The approximate position of the membrane is shown based on the hydrophobic and positive charge regions of the protein surface. The N-terminus is expected to be on the extracellular side of the membrane with the C-terminus in the cytoplasm. The formyl peptide binding site is located in a pocket formed in the middle of the transmembrane helices. Residues predicted to bind formyl peptides are found lining this pocket.

**Fig 10:**
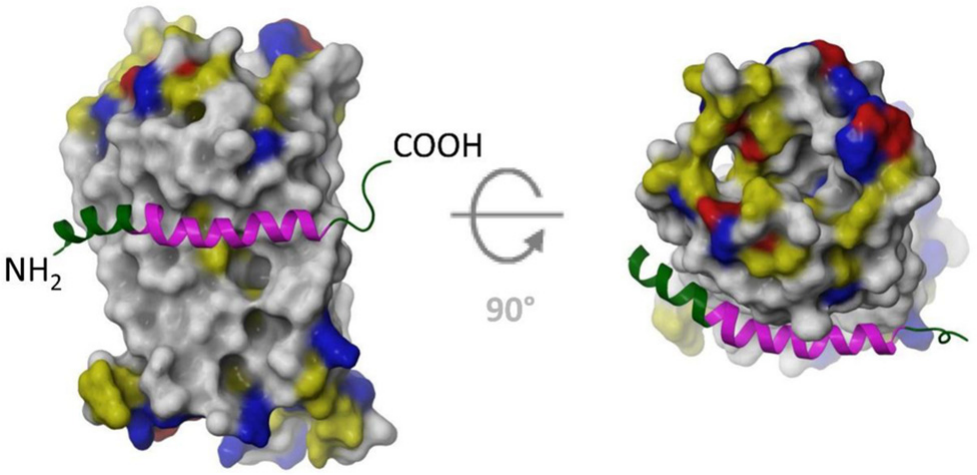
Docking of LL-37 and GI-20 to FPR-2—The docked peptide is shown as a green ribbon, with the residues of GI-20 highlighted in magenta. **–** LL-37 docks to the outside of the receptor, with the hydrophobic face of the LL-37 amphipathic helix. The hydrophobic face of LL-37 interacts with the hydrophobic residues of the transmembrane helices of FPR-2 in this model. The polar side of LL-37 consists of mostly positively charged residues, which could favorably interact with the polar headgroups of the membrane. Note that the area of contact of LL-37 with the receptor mostly coincides with GI-20.

Our studies provided the basis for development of a homology model for the interaction of LL-37 with FPR-2. We developed a homology model of FPR-2 (whose crystal structure has not been established), using known structures of related seven transmembrane spanning G-protein coupled receptors (see Fig 9). Using this model we performed docking experiments with the known NMR structure of LL-37. The best fit model is shown in Fig 10. Note that LL-37 did not bind to the formyl peptide receptor binding pocket but rather to the membrane interface of the receptor. Based on this model the ability of LL-37 to activate the receptor would depend on allosteric changes in the receptor. The model fits well with our findings in that the area of contact of LL-37 with the receptor overlaps with that of GI-20. It is well established that LL-37 interacts with membranes and our model is consistent with this and also accounts for the ability of GI-20 to activate this receptor. The failure of LL-23 to activate the receptor suggests that amino acids 24–32 may make key contacts with the receptor (since these are included in GI-20 but not in LL-23). Also amino acids 1–12 do not seem to be necessary for receptor interaction. Amino acids 1–4 do not contact the receptor surface in our model. Initially we were surprised that the D-isomer of GI-20 retained the respiratory burst activating effect, however, this also is consistent with our model. If LL-37 were interacting in a stereospecific manner with the formyl peptide binding domain of the receptor then the D-isomer would not be expected to retain activity. However, the D-isomer is expected to fold as a left handed helix, with similar amphipathic nature and curvature as the L-isomer (i.e. a complete mirror image). Given this then the interaction with FPR-2 could be retained based on our model.

The model of course requires further testing. Lacking a formal structure of the any formyl peptide receptor it is not possible to take a receptor mutagenesis approach. Singh et al found that a chimera containing amino acids 1–29 of LL-37 and 30–37 of CRAMP retained the ability of LL-37 to activate responses to poly I:C in airway epithelial cells, whereas a construct in which amino acids 30–37 of LL-37 were substituted into CRAMP did not [45]. This suggests that at a minimum amino acids up to 29 are needed for FPR-2 interaction and that the divergence between LL-37 (or GI-20) and CRAMP leads to loss of the ability to activate FPR-2 in the presence of IAV. As shown in Table 1, CRAMP is not highly homologous with LL-37. In the region shared with GI-20 there are only 8 conserved amino acids. Hence, it is possible that the divergent amino acids play and important role in interaction of GI-20 with FPR-2. Use of variously modified forms of LL-37 or GI-20 is an approach that we plan to take to validate our model.

**Table 1:** Comparison of sequences of peptides used in this study.

Peptide	Peptide Sequence^a^
LL-37	LLGDFFRKSKEKIGKEFKRIVQRIKDFLRNLVPRTES
LL-23	LLGDFFRKSKEKIGKEFKRIVQR
LL-23V9	LLGDFFRK**V**KEKIGKEFKRIVQR
GI-20	**GI**KEFKRIVQRIKDFLRNLV
FK-13	FKRIVQRIKDFLR
KR-12	KRIVQRIKDFLR
CRAMP	GLLRKGGEKIGEKLKKIGQKIKNFFQKLVPQPEQ

^**a**^ The amino acids which have been changed in LL-23V9 and GI-20 (as compared to LL-37) are shown in bold. Amino acids that are the same in LL-37 and CRAMP are underlined in the CRAMP sequence.

There are some caveats with regard to our model that remain hard to fully explain. There is the question of how WDW4 which binds to the formyl peptide binding pocket can inhibit responses elicited by LL-37 or GI-20 when these bind to a different domain of the protein. We can only speculate that binding of WDW4 in some way locks the receptor so that it cannot undergo the allosteric change mediated by LL-37 or GI-20. LL-37 and GI-20 do not inhibit IAV’s hemagglutination activity and it is hemagglutinin binding that mediates direct activation of neutrophils by IAV [47]. We propose that activation by the complex of IAV with LL-37 or GI-20 involves two pathways (through engagement of FPR-2 and engagement of distinct IAV receptors). We have similar findings with regard to enhanced neutrophil respiratory burst activation by IAV complexed with surfactant protein D or other collectins [48]. This would explain why the activation is only partially inhibited by pertussis toxin (which blunts the FPR-2 mediated component of the response). Hopefully, further research will be able to clarify these issues. Note that lipoxin A, resolvin, serum amyloid protein and annexin A-1 also bind to FPR-2 resulting variably in anti- and pro-inflammatory effects [44, 49, 50]. Hence, a better understanding of the possibly diverse mechanisms through which the receptor can be triggered is very important.

We have previously shown that GI-20 retains the antiviral activity of LL-37 and has the ability to inhibit pandemic IAV while LL-37 does not [51]. In this paper we show that GI-20 modulates neutrophil activation in a manner similar to LL-37. GI-20 or other derivatives of the central helix of LL-37 appear, therefore, to be of interest for development as therapeutics.

## References

[1] Tripathi S, White MR, Hartshorn KL. The amazing innate immune response to influenza A virus infection. Innate Immun. 2015;21(1):73–98. 10.1177/1753425913508992 . 24217220

[2] Oppenheim JJ, Yang D. Alarmins: chemotactic activators of immune responses. Curr Opin Immunol. 2005;17(4):359–65. . 15955682 10.1016/j.coi.2005.06.002

[3] Gwyer Findlay E, Currie SM, Davidson DJ. Cationic Host Defence Peptides: Potential as Antiviral Therapeutics. BioDrugs. 2013 . 23649937 10.1007/s40259-013-0039-0 PMC3775153

[4] Gong T, Jiang Y, Wang Y, Yang D, Li W, Zhang Q, et al Recombinant mouse beta-defensin 2 inhibits infection by influenza A virus by blocking its entry. Arch Virol. 2010;155(4):491–8. 10.1007/s00705-010-0608-1 20195655

[5] Jiang Y, Wang Y, Kuang Y, Wang B, Li W, Gong T, et al Expression of mouse beta-defensin-3 in MDCK cells and its anti-influenza-virus activity. Arch Virol. 2009;154(4):639–47. 10.1007/s00705-009-0352-6 19301094

[6] Salvatore M, Garcia-Sastre A, Ruchala P, Lehrer RI, Chang T, Klotman ME. alpha-Defensin inhibits influenza virus replication by cell-mediated mechanism(s). J Infect Dis. 2007;196(6):835–43. . 17703413 10.1086/521027

[7] Leikina E, Delanoe-Ayari H, Melikov K, Cho MS, Chen A, Waring AJ, et al Carbohydrate-binding molecules inhibit viral fusion and entry by crosslinking membrane glycoproteins. Nat Immunol. 2005;6(10):995–1001. . 16155572 10.1038/ni1248

[8] Tripathi S, Tecle T, Verma A, Crouch E, White M, Hartshorn KL. The human cathelicidin LL-37 inhibits influenza A viruses through a mechanism distinct from that of surfactant protein D or defensins. J Gen Virol. 2013;94(Pt 1):40–9. 10.1099/vir.0.045013-0 23052388 PMC3542722

[9] Doss M, White MR, Tecle T, Gantz D, Crouch EC, Jung G, et al Interactions of alpha-, beta-, and theta-defensins with influenza A virus and surfactant protein D. J Immunol. 2009;182(12):7878–87. 10.4049/jimmunol.0804049 19494312

[10] Hartshorn KL, White MR, Tecle T, Holmskov U, Crouch EC. Innate defense against influenza A virus: activity of human neutrophil defensins and interactions of defensins with surfactant protein D. J Immunol. 2006;176(11):6962–72. . 16709857 10.4049/jimmunol.176.11.6962

[11] Sweet C, Smith H. Pathogenicity of influenza virus. Microbiol Rev. 1980;44:303–30. 6991901 10.1128/mr.44.2.303-330.1980 PMC373180

[12] Barlow PG, Svoboda P, Mackellar A, Nash AA, York IA, Pohl J, et al Antiviral activity and increased host defense against influenza infection elicited by the human cathelicidin LL-37. PLoS One. 2011;6(10):e25333 10.1371/journal.pone.0025333 22031815 PMC3198734

[13] Gaudreault E, Gosselin J. Leukotriene B4 induces release of antimicrobial peptides in lungs of virally infected mice. J Immunol. 2008;180(9):6211–21. . 18424743 10.4049/jimmunol.180.9.6211

[14] Doss M, White MR, Tecle T, Hartshorn KL. Human defensins and LL-37 in mucosal immunity. J Leukoc Biol. 2010;87:79–92. 10.1189/jlb.0609382 19808939 PMC7167086

[15] Li HN, Barlow PG, Bylund J, Mackellar A, Bjorstad A, Conlon J, et al Secondary necrosis of apoptotic neutrophils induced by the human cathelicidin LL-37 is not proinflammatory to phagocytosing macrophages. J Leukoc Biol. 2009;86(4):891–902. 10.1189/jlb.0209050 19581375 PMC2791992

[16] Tecle T, White MR, Gantz D, Crouch EC, Hartshorn KL. Human neutrophil defensins increase neutrophil uptake of influenza A virus and bacteria and modify virus-induced respiratory burst responses. J Immunol. 2007;178(12):8046–52. . 17548642 10.4049/jimmunol.178.12.8046

[17] Tripathi S, White MR, Wang G, Hartshorn K. LL-37 modulates human phagocyte responses to influenza A virus. 2014; 96: 931–8. 10.1189/jlb.4A1113-604RR PMC4197563 25082153

[18] Mookherjee N, Wilson HL, Doria S, Popowych Y, Falsafi R, Yu JJ, et al Bovine and human cathelicidin cationic host defense peptides similarly suppress transcriptional responses to bacterial lipopolysaccharide. J Leukoc Biol. 2006;80(6):1563–74. . 16943385 10.1189/jlb.0106048

[19] De Y, Chen Q, Schmidt AP, Anderson GM, Wang JM, Wooters J, et al LL-37, the neutrophil granule- and epithelial cell-derived cathelicidin, utilizes formyl peptide receptor-like 1 (FPRL1) as a receptor to chemoattract human peripheral blood neutrophils, monocytes, and T cells. J Exp Med. 2000;192(7):1069–74. . 11015447 10.1084/jem.192.7.1069 PMC2193321

[20] Mookherjee N, Lippert DN, Hamill P, Falsafi R, Nijnik A, Kindrachuk J, et al Intracellular receptor for human host defense peptide LL-37 in monocytes. J Immunol. 2009;183(4):2688–96. 10.4049/jimmunol.0802586 19605696

[21] Zhang Z, Cherryholmes G, Chang F, Rose DM, Schraufstatter I, Shively JE. Evidence that cathelicidin peptide LL-37 may act as a functional ligand for CXCR2 on human neutrophils. Eur J Immunol. 2009;39(11):3181–94. 10.1002/eji.200939496 19750480 PMC3076219

[22] Tjabringa GS, Aarbiou J, Ninaber DK, Drijfhout JW, Sorensen OE, Borregaard N, et al The antimicrobial peptide LL-37 activates innate immunity at the airway epithelial surface by transactivation of the epidermal growth factor receptor. J Immunol. 2003;171(12):6690–6. . 14662872 10.4049/jimmunol.171.12.6690

[23] Nagaoka I, Tamura H, Hirata M. An antimicrobial cathelicidin peptide, human CAP18/LL-37, suppresses neutrophil apoptosis via the activation of formyl-peptide receptor-like 1 and P2X7. J Immunol. 2006;176(5):3044–52. . 16493063 10.4049/jimmunol.176.5.3044

[24] Murakami M, Lopez-Garcia B, Braff M, Dorschner RA, Gallo RL. Postsecretory processing generates multiple cathelicidins for enhanced topical antimicrobial defense. J Immunol. 2004;172(5):3070–7. . 14978112 10.4049/jimmunol.172.5.3070

[25] Dannehl C, Gutsmann T, Brezesinski G. Surface activity and structures of two fragments of the human antimicrobial LL-37. Colloids and surfaces B, Biointerfaces. 2013;109:129–35. 10.1016/j.colsurfb.2013.03.030 . 23624281

[26] Rico-Mata R, De Leon-Rodriguez LM, Avila EE. Effect of antimicrobial peptides derived from human cathelicidin LL-37 on Entamoeba histolytica trophozoites. Experimental parasitology. 2013;133(3):300–6. 10.1016/j.exppara.2012.12.009 . 23274811

[27] Wang G, Elliott M, Cogen AL, Ezell EL, Gallo RL, Hancock RE. Structure, dynamics, and antimicrobial and immune modulatory activities of human LL-23 and its single-residue variants mutated on the basis of homologous primate cathelicidins. Biochemistry. 2012;51(2):653–64. 10.1021/bi2016266 22185690 PMC3302206

[28] Wang G. Structures of human host defense cathelicidin LL-37 and its smallest antimicrobial peptide KR-12 in lipid micelles. J Biol Chem. 2008;283(47):32637–43. 10.1074/jbc.M805533200 18818205

[29] Wang G, Hanke ML, Mishra B, Lushnikova T, Heim CE, Chittezham Thomas V, et al Transformation of human cathelicidin LL-37 into selective, stable, and potent antimicrobial compounds. ACS chemical biology. 2014;9(9):1997–2002. 10.1021/cb500475y 25061850 PMC4168778

[30] Jacob B, Park IS, Bang JK, Shin SY. Short KR-12 analogs designed from human cathelicidin LL-37 possessing both antimicrobial and antiendotoxic activities without mammalian cell toxicity. J Pept Sci. 2013;19(11):700–7. 10.1002/psc.2552 . 24105706

[31] Wang G, Mishra B, Epand RF, Epand RM. High-quality 3D structures shine light on antibacterial, anti-biofilm and antiviral activities of human cathelicidin LL-37 and its fragments. Biochim Biophys Acta. 2014;1838(9):2160–72. 10.1016/j.bbamem.2014.01.016 24463069 PMC4082733

[32] Tripathi S, Wang G, White M, Qi L, Taubenberger J, Hartshorn KL. Antiviral Activity of the Human Cathelicidin, LL-37, and Derived Peptides on Seasonal and Pandemic Influenza A Viruses. PLoS One. 2015;10(4):e0124706 10.1371/journal.pone.0124706 25909853 PMC4409069

[33] Hartshorn KL, Collamer M, Auerbach M, Myers JB, Pavlotsky N, Tauber AI. Effects of influenza A virus on human neutrophil calcium metabolism. J Immunol. 1988;141(4):1295–301. 3135328

[34] Hartshorn KL, White MR, Shepherd V, Reid K, Jensenius JC, Crouch EC. Mechanisms of anti-influenza activity of surfactant proteins A and D: comparison with serum collectins. Am J Physiol. 1997;273(6 Pt 1):L1156–66. . 9435570 10.1152/ajplung.1997.273.6.L1156

[35] Hartshorn KL, Collamer M, White MR, Schwartz JH, Tauber AI. Characterization of influenza A virus activation of the human neutrophil. Blood. 1990;75(1):218–26. 2153030

[36] Hartshorn KL, White MR, Voelker DR, Coburn J, Zaner K, Crouch EC. Mechanism of binding of surfactant protein D to influenza A viruses: importance of binding to haemagglutinin to antiviral activity. Biochem J. 2000;351 Pt 2:449–58. . 11023831 PMC1221381

[37] Roy A, Kucukural A, Zhang Y. I-TASSER: a unified platform for automated protein structure and function prediction. Nature protocols. 2010;5(4):725–38. 10.1038/nprot.2010.5 20360767 PMC2849174

[38] Tan Q, Zhu Y, Li J, Chen Z, Han GW, Kufareva I, et al Structure of the CCR5 chemokine receptor-HIV entry inhibitor maraviroc complex. Science. 2013;341(6152):1387–90. 10.1126/science.1241475 24030490 PMC3819204

[39] Fenalti G, Giguere PM, Katritch V, Huang XP, Thompson AA, Cherezov V, et al Molecular control of delta-opioid receptor signalling. Nature. 2014;506(7487):191–6. 10.1038/nature12944 24413399 PMC3931418

[40] Cherezov V, Rosenbaum DM, Hanson MA, Rasmussen SG, Thian FS, Kobilka TS, et al High-resolution crystal structure of an engineered human beta2-adrenergic G protein-coupled receptor. Science. 2007;318(5854):1258–65. 10.1126/science.1150577 17962520 PMC2583103

[41] Comeau SR, Kozakov D, Brenke R, Shen Y, Beglov D, Vajda S. ClusPro: performance in CAPRI rounds 6–11 and the new server. Proteins. 2007;69(4):781–5. 10.1002/prot.21795 . 17876812

[42] Hartshorn KL, Wright J, Collamer MA, White MR, Tauber AI. Human neutrophil stimulation by influenza virus: relationship of cytoplasmic pH changes to cell activation. Am J Physiol. 1990;258(6 Pt 1):C1070–6. . 2113768 10.1152/ajpcell.1990.258.6.C1070

[43] Tripathi S, Verma A, Kim EJ, White MR, Hartshorn KL. LL-37 modulates human neutrophil responses to influenza A virus. J Leukoc Biol. 2014 10.1189/jlb.4A1113-604RR . 25082153 PMC4197563

[44] Wan M, Godson C, Guiry PJ, Agerberth B, Haeggstrom JZ. Leukotriene B4/antimicrobial peptide LL-37 proinflammatory circuits are mediated by BLT1 and FPR2/ALX and are counterregulated by lipoxin A4 and resolvin E1. FASEB J. 2011;25(5):1697–705. 10.1096/fj.10-175687 . 21307335

[45] Singh D, Qi R, Jordan JL, San Mateo L, Kao CC. The human antimicrobial peptide LL-37, but not the mouse ortholog, mCRAMP, can stimulate signaling by poly(I:C) through a FPRL1-dependent pathway. J Biol Chem. 2013;288(12):8258–68. 10.1074/jbc.M112.440883 23386607 PMC3605644

[46] Douda DN, Khan MA, Grasemann H, Palaniyar N. SK3 channel and mitochondrial ROS mediate NADPH oxidase-independent NETosis induced by calcium influx. Proc Natl Acad Sci U S A. 2015;112(9):2817–22. 10.1073/pnas.1414055112 . 25730848 PMC4352781

[47] Daigneault DE, Hartshorn KL, Liou LS, Abbruzzi GM, White MR, Oh SK, et al Influenza A virus binding to human neutrophils and cross-linking requirements for activation. Blood. 1992;80(12):3227–34. . 1334733

[48] Hartshorn KL, Reid KB, White MR, Jensenius JC, Morris SM, Tauber AI, et al Neutrophil deactivation by influenza A viruses: mechanisms of protection after viral opsonization with collectins and hemagglutination-inhibiting antibodies. Blood. 1996;87(8):3450–61. . 8605364

[49] Buss NA, Gavins FN, Cover PO, Terron A, Buckingham JC. Targeting the annexin 1-formyl peptide receptor 2/ALX pathway affords protection against bacterial LPS-induced pathologic changes in the murine adrenal cortex. FASEB J. 2015 10.1096/fj.14-268375 . 25818588

[50] Le Y, Yang Y, Cui Y, Yazawa H, Gong W, Qiu C, et al Receptors for chemotactic formyl peptides as pharmacological targets. Int Immunopharmacol. 2002;2(1):1–13. . 11789660 10.1016/s1567-5769(01)00150-3

[51] Tripathi S, Wang G, White M, Qi L, Taubenberger J, Hartshorn K. Antiviral activity of the human cathelicidin, LL-37, and derived peptides on seasonal and pandemic influenza A virus. PLOS One. 2015; e0214706. 10.1371/journal.pone.0124706 PMC4409069 25909853

